# Association between reading and depression in Chinese adults

**DOI:** 10.1097/MD.0000000000032486

**Published:** 2022-12-23

**Authors:** Degong Pan, Zhiqin Hai, Xiao Yang, Shulan He, Xiaojun Li, Jiangping Li

**Affiliations:** a Department of Epidemiology and Health Statistics, School of Public Health and Management, Ningxia Medical University, Yinchuan, Ningxia Hui Autonomous Region, China; b Neuroscience Center, General Hospital of Ningxia Medical University, Key Laboratory of Craniocerebral Diseases of Ningxia Hui Autonomous Region, Yinchuan, China; c Yijinhuoluo Disease Control and Prevention Center, Erdos, Inner Mongolia, China; d Research Center of Health Big Data, Key Laboratory of Environmental Factors and Chronic Disease Control, Yinchuan, Ningxia Hui Autonomous Region, China.

**Keywords:** China, depression, reading, the elderly

## Abstract

Qualitative evidences have shown that having the habit of reading might be beneficial for mental health. The present study aims to examine the relationship between reading and depression. National cross-sectional survey data of adults aged >40 years in mainland China were used. The Center for Epidemiological Studies Depression Scale questionnaire was utilized to detect depression status. Multilevel binary logistic and linear regression models were employed to reveal the association, and restricted cubic spline with 4 knots was adopted to describe the non-linear association of reading quantity and depression. The prevalence of depression was 13.02% in the target population. It was found that the habit of reading was negatively associated with depression, the odds ratio was 0.809 (95% confidence interval: 0.657–0.997). Diverse association between reading and depression was observed in different age groups, and a significant association was identified among the elderly, but not in the middle-aged population. Restricted cubic spline showed several books read per year might lower the risk of depression and 20-items Center for Epidemiological Studies Depression Scale score. A lower prevalence of depression was observed in the target population. The habit of reading was negatively associated with depression. Age-specific association was observed. It is worth paying attention to the reading habit that could be beneficial in the elderly for mental health intervention, but it needs to be confirmed by experimental study.

## 1. Introduction

Depression has become a widely prevalent public health concern related with dementia,^[1]^ hypersomnia,^[2]^ sleep disturbance,^[3]^ cardiovascular disease,^[4]^ obesity,^[5]^ and even suicide,^[6,7]^ which has been identified as a burden on both individuals and societies. In China, the lifetime prevalence of depression was 6.9% and the 12-month prevalence was 3.6%, and compared with most high-income countries, the proportion of people with depression receiving mental health services is low.^[8]^ Mental health problem is a staged process, a study revealed that middle-aged and elderly individuals are susceptible to various mental health conditions.^[9]^ Therefore, it is of great social significance to pay attention to depression among middle-aged and elderly people.

Unfortunately, there has been an increasing trend in the social and financial burden of mental health management in recent years. Numerous medical methods have been used to treat depression, nonetheless, many side effects exist.^[10–12]^ It is, therefore, necessary to prevent and alleviate depression before further deterioration. The community care, including hygiene intervention and living habits, such as reading, is commonly regarded as a valid treatment for depression in China, which is also defined as part of a goal in health programs for the elderly.

Reading has been posited as a cost-effective way of improving mood,^[13]^ because wisdom is the primary foundation of health that guides individuals to acquire a philosophy of life to regulate their health behaviors.^[14]^ It may assist individuals in modulating their emotions and coping with stress and have a significant association with depression. However, previous studies on reading and depression had small sample sizes and were unrepresentative.^[15,16]^ Thus, further large population-based survey evidence is required to confirm this claim. In the present study, we wondered whether there was a relationship between reading habits and depression in middle and elderly adults using China Family Panel Studies (CFPS) survey data.

## 2. Methods

### 2.1. Study sample

Data was obtained from the CFPS, funded by the 985 Program of Peking University, and carried out by the Institute of Social Science Research of Peking University. The CFPS sample was designed to be multi-stage, to reduce the operational costs of the survey and represent the heterogeneity of social contexts.^[17]^ This is an ongoing nationally representative, longitudinal, household, biennial survey, covering 95% of the Chinese population, from 25 provinces.^[18]^ It reflects the transition of social, financial, educational, and health circumstances in the population, from 3 levels: individual, family, and community. The dataset of the 2016 survey wave was selected due to health conditions, and demographics were collected with reading information simultaneously. A total of 21916 subjects over the age of 40 were selected in current study.

### 2.2. Measurement of depression

Depression was measured using the 20-items Center for Epidemiological Studies Depression Scale (CES-D_20_). Respondents answered items on the frequency of their symptoms on a 4-point scale where 1 referred to less than a day; 2 referred to 1 to 2 days; 3 referred to 3 to 4 days, and 4 referred to 5 to 7 days, in terms of a time frame of the previous week.^[19,20]^ The scale was originally developed to measure depressive symptomatology in the general population, and the items, as experienced in the past 7 days, were defined by the American Psychiatric Association Diagnostic and Statistical Manual for a major depressive episode.^[21]^ The total score of the scale was 80, the lowest possible score was 20, and based on the initial purpose, the recommended threshold value was defined as 42.^[19]^ Hence, scores >42 were defined as depression.

### 2.3. Reading definition

Two questions were used to evaluate the reading status. The first was a yes/no question: “Have you read a book in the past 12 months that was not for the purpose of work or exam (including e-books, but not magazines)?” Participants who answered yes were assigned to a reading habit group. The second was: “How many books (including e-books, but not magazines) have you read in the past 12 months, excluding reading for work and exam purposes?” The number of readings might relate to a good reading habit.

### 2.4. Covariates

Covariates extracted as place of residence (urban or rural), age, gender, education level (illiterate, primary school, junior high school, senior high school or above), family size, ethnic group (Han, and others), marital status (never married, married/cohabitation, divorced/widowed), number of children, income status that was measured in the question “what is your income level in this area?” to avoid underestimations, social status that was measured through the question “What do you think of your social status in local region?,” and life satisfaction that was measured by the question “How would you rate your life satisfaction?.” These indices were evaluated on a 5-point Likert scale, where 5 represented the most advanced status. Health-related variables such as BMI group (underweight, BMI < 18.5; normal, 18.5 ≤ BMI < 24.0; overweight, 24.0 ≤ BMI < 28.0; obesity, BMI ≥ 28.0) were also investigated. Self-rated health was measured through a 5-point scale, in terms of “excellent,” “very good,” “good,” “fair,” and “poor health”. Health changes were measured using a 3-point scale, involving the choices “better,” “no change,” and “worse”. This was framed as a question wherein respondents were asked to rate their current health status, compared to that of the previous year. Chronic diseases were investigated by the yes/no question: “Were you diagnosed with a chronic disease in the past six months by a doctor?.” The last health-related variable was physical discomfort experienced during the previous 2 weeks (yes/no options). Four lifestyle related items were considered in the present study: smoking, which was evaluated by the yes/no question “Have you smoked in the past month?”; drinking was measured by the yes/no question “Have you been drinking alcohol 3 times a week in the past month?”; the yes/no question of “Do you have a habit of taking a nap?” was used to collect information on noon break habits; dinner frequency with family per week (quantitative variable) was measured through the question “How many times in a week do you have dinner with your family?.”

### 2.5. Analytic strategy

Analyses were conducted using Stata MP Version 15.0 (STATA Corporation, College Station, TX) software. The mean and standard deviation were used to describe quantitative variables, while counts and percentages were employed to depict qualitative variables. Two independent sample *t* tests and chi-square tests examined the difference between depression status and reading habits. The visual association between depression and the number of readings was described by restrict cubic spline with 4 knots that all of covariates were controlled. A multilevel, mixed-effects logistic regression model was used to determine the relationship between depression and reading habits. The region surveyed and family unit were considered as multilevel proxies.

Five models were set: Model 0 was defined as an empty model that just included reading habit or number of readings as an explanation variable. Model 1 was based on Model 0, with demographic information added as controlled variables. In Model 2, socioeconomic variables were considered based on Model 1. Model 3 was defined as Model 2 plus health-related variables. Finally, the full model established as Model 4 consisted of lifestyle information encompassed within Model 3. In addition, age was demarcated into 2 groups (middle-aged adults aged from 41–65 and the elderly aged >65) to identify the age-specific association.

## 3. Results

### 3.1. Basic information description

The characteristics of the sample according to the depression and reading habits are displayed in Table 1. A fair number of baseline characters showed significant distribution in depression status and reading habits group simultaneously.

**Table 1:** Basic information distribution of depression & reading habit.

Variables	Depression		*P*	Reading habit		*P*
	No	Yes		No	Yes	
Demographic information						
Place of residence			<.001			<.001
Rural	8894 (84.66)	1611 (15.34)		9536 (90.76)	971 (9.24)	
Urban	8466 (89.58)	985 (10.42)		7659 (81.03)	1793 (18.97)	
Gender			<.001			<.001
Female	8394 (83.91)	1609 (16.09)		9021 (90.16)	984 (9.84)	
Male	9036 (90.04)	999 (9.96)		8241 (82.11)	1795 (17.89)	
Ethnic			<.001			<.001
Han	16,131 (87.30)	2346 (12.70)		15,859 (85.82)	2621 (14.18)	
Others	1245 (83.06)	254 (16.94)		1346 (89.79)	153 (10.21)	
Education level			<.001			<.001
Illiterate	5795 (81.33)	1330 (18.67)		6942 (97.42)	184 (2.58)	
Primary school	3856 (87.18)	567 (12.82)		3970 (89.76)	453 (10.24)	
Junior high school	4280 (91.49)	398 (8.51)		3801 (81.25)	877 (18.75)	
Senior high school or above	2658 (92.90)	203 (7.10)		1735 (60.64)	1126 (39.36)	
Marital status			<.001			<.001
Never married	169 (72.84)	63 (27.16)		191 (82.33)	41 (17.67)	
Married/Cohabitation	15,599 (88.62)	2004 (11.38)		15,084 (85.68)	2521 (14.32)	
Divorced/Widowed	1661 (75.43)	541 (24.57)		1986 (90.15)	217 (9.85)	
Age (mean ± SD)	56.94 ± 10.70	58.95 ± 11.14	<.001	57.65 ± 10.73	54.41 ± 10.64	<.001
Number of children (mean ± SD)	2.09 ± 1.14	2.35 ± 1.33	<.001	2.19 ± 1.19	1.74 ± 1.00	<.001
Family size (mean ± SD)	4.09 ± 1.98	3.85 ± 2.01	<.001	4.11 ± 2.01	3.73 ± 1.75	<.001
Socioeconomic level						
Income status (mean ± SD)	2.44 ± 1.03	2.05 ± 1.13	<.001	2.37 ± 1.07	2.51 ± 0.95	<.001
Social status (mean ± SD)	2.95 ± 1.09	2.68 ± 1.27	<.001	2.91 ± 1.13	2.93 ± 1.03	.476
Life satisfaction (mean ± SD)	3.76 ± 1.04	3.10 ± 1.30	<.001	3.68 ± 1.11	3.67 ± 1.03	.827
Health related						
BMI group			<.001			<.001
Underweight	1326 (76.34)	411 (23.66)		1600 (92.06)	138 (7.94)	
Normal	9302 (87.14)	1373 (12.86)		9185 (86.04)	1490 (13.96)	
Overweight	5057 (90.05)	559 (9.95)		4717 (83.99)	899 (16.01)	
Obesity	1526 (87.90)	210 (12.10)		1505 (86.69)	231 (13.31)	
Self-rated health			<.001			<.001
Excellent	1823 (96.00)	76 (4.00)		1638 (86.17)	263 (13.83)	
Very good	2440 (95.20)	123 (4.80)		2230 (87.01)	333 (12.99)	
Good	6240 (92.28)	522 (7.72)		5497 (81.29)	1265 (18.71)	
Fair	3916 (88.70)	499 (11.30)		3891 (88.13)	524 (11.87)	
Poor	3010 (68.44)	1388 (31.56)		4004 (91.04)	394 (8.96)	
Health change			<.001			<.001
Better	1641 (89.87)	185 (10.13)		1540 (84.29)	287 (15.71)	
No change	9499 (93.33)	679 (6.67)		8579 (84.29)	1599 (15.71)	
Worse	6289 (78.29)	1744 (21.71)		7142 (88.89)	893 (11.11)	
Discomfort in past 2 wk			<.001			.005
No	12,204 (92.68)	964 (7.32)		11,279 (85.64)	1891 (14.36)	
Yes	5224 (76.06)	1644 (23.94)		5981 (87.07)	888 (12.93)	
Chronic disease			<.001			.813
No	13,887 (89.95)	1552 (10.05)		13,304 (86.17)	2136 (13.83)	
Yes	3540 (77.06)	1054 (22.94)		3953 (86.03)	642 (13.97)	
Life style						
Smoking			<.001			<.001
No	12,127 (86.03)	1970 (13.97)		12,284 (87.12)	1816 (12.88)	
Yes	5302 (89.26)	638 (10.74)		4977 (83.79)	963 (16.21)	
Drinking			<.001			<.001
No	14,262 (85.97)	2328 (14.03)		14,420 (86.90)	2173 (13.10)	
Yes	3168 (91.88)	280 (8.12)		2842 (82.42)	606 (17.58)	
Noon break habit			<.001			<.001
Yes	9532 (87.79)	1326 (12.21)		9151 (84.27)	1708 (15.73)	
No	7897 (86.03)	1282 (13.97)		8110 (88.33)	1071 (11.67)	
Diner time with family per wk (mean ± SD)	6.33 ± 1.79	6.09 ± 2.13	<.001	6.34 ± 1.80	6.02 ± 2.02	<.001

BMI = body mass index, SD = standard deviation.

A univariate analysis revealed that the CES-D_20_ score in the reading habit group was significantly lower than that in the non-reading group (Table 2). The prevalence of depression was 13.82% in the non-reading group, as opposed to 7.99% in the reading group. Our results indicated that only 13.87% of participants had a habit of reading.

**Table 2:** Differential test of depression between reading habit.

Variables	Reading habit		*t*/*χ*^2^	*P*
	No	Yes		
CES-D_20_ (mean ± SD)	33.40 ± 8.85	31.34 ± 7.74	11.548	<.001
Depression (n, %)			72.013	<.001
No	14,873 (86.18)	2557 (92.01)		
Yes	2386 (13.82)	222 (7.99)		

CES-D_20_ = 20-items Center for Epidemiological Studies Depression Scale, SD = standard deviation.

### 3.2. The prevalence of depression

Using the CES-D_20_ questionnaire, we identified that the prevalence of depression in our sample was 13.02%, which indicated that approximately 13 in 100 participants suffered from depression.

### 3.3. Association of CES-D_20_ score and the number of readings

**Figure 1.**
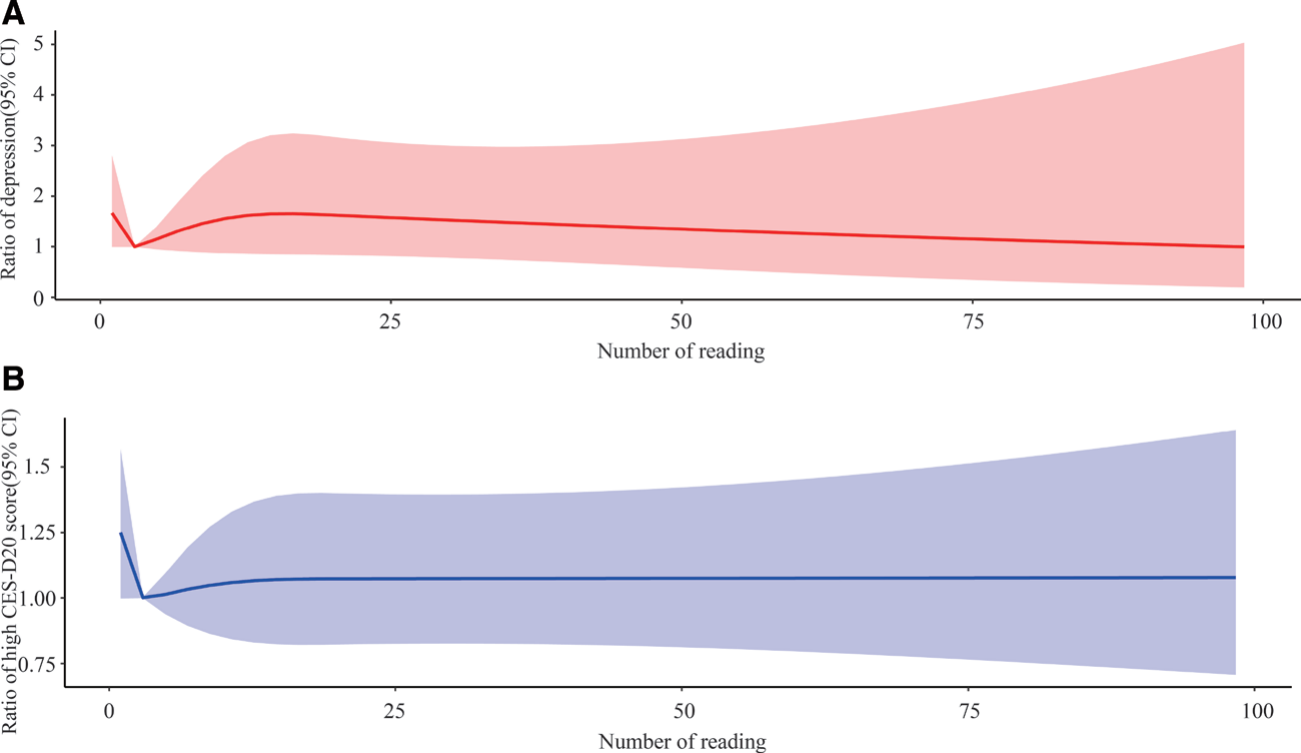
Association between number of readings and depression, CES-D_20_ score that controlling for place of residence, gender, ethnic, education level, marital status, age, number of children, family size, income status, social status, life satisfaction, BMI group, self-rated health, health change, discomfort in past 2 weeks, chronic disease, smoking, drinking, noon break habit, and diner time with family per week. Figure 1A: association between number of readings and depression; Figure 1B: association between number of readings and CES-D_20_ score. CES-D_20_ = 20-items Center for Epidemiological Studies Depression Scale.

The association of the CES-D_20_ score and the number of readings, can be seen in Figure 1B, implies that with fewer number of readings, the CES-D_20_ score was slightly higher, and while the number reached about 4 books per year the CES-D_20_ score reached the lowest, however, when the number increased the CES-D score raised slowly and gradually stabilized.

### 3.4. Association between reading and depression

The relationship between reading and depression is displayed in Table 3. A robust effect was found by means of 5 regression models. Reading habits were negatively associated with depression, with an odds ratio (OR) of 0.809 in full model.

**Table 3:** Association between reading habit and depression, CES-D_20_ score with ORs/coefficient and 95% CIs.

Model	Depression†		CES-D_20_ score‡	
	OR	95% CI	*β*	95% CI
Model0	0.499***	0.422, 0.591	−1.757***	−2.094, −1.419
Model1	0.808**	0.671, 0.974	−0.337*	−0.697,0.022
Model2	0.856	0.708, 1.034	−0.159	−0.505,0.187
Model3	0.799**	0.655, 0.975	−0.297*	−0.621,0.028
Model4	0.809**	0.657, 0.997	−0.279*	−0.607,0.049

Model 0 contains depression category. Model 1 = Model0 + demographic variables. Model 2 = Model1 + socioeconomic level information. Model 3 = Model2 + health related indexes. Model 4 is the full model that contains all confounders and predictors.

CES-D_20_ = 20-items Center for Epidemiological Studies Depression Scale, CI = confidence interval, OR = odds ratio, β = linear regression coefficient.

† Multilevel binary logistic regression model was conducted.

‡ Multilevel linear regression model was conducted.

* *P* < .1.

** *P* < .05.

*** *P* < .01.

Evidence derived from our sample did not reveal a significant association between the number of readings with depression categories, and CES-D_20_ score (Table 4). However, as shown in Figure 1A, the trend was similar as the relationship between the number of readings and the CES-D_20_ score, for example, when the number of readings is about 4 books per year, the risk of depression reached the lowest; And the risk of depression was increased with the number of readings increased and then, when the number of readings were >15 the risk of depression was reduced instead.

**Table 4:** Association between number of readings and depression, CES-D_20_ score with ORs/coefficient and 95% CIs.

Model	Depression†		CES-D_20_ score‡	
	OR	95% CI	*β*	95% CI
Model0	0.974	0.923, 1.028	−0.017**	−0.031, −0.002
Model1	0.965	0.918, 1.015	−0.011	−0.027, 0.005
Model2	0.997	0.982, 1.012	−0.006	−0.021, 0.009
Model3	0.996	0.982, 1.010	−0.008	−0.022, 0.005
Model4	0.999	0.986, 1.012	−0.008	−0.021, 0.006

Model 0 contains depression category; Model 1 = Model0 + demographic variables; Model 2 = Model1 + socioeconomic level information; Model 3 = Model2 + health related indexes; Model 4 is the full model that contains all confounders and predictors.

CES-D_20_ = 20-items Center for Epidemiological Studies Depression Scale, CI = confidence interval, OR = odds ratio, *β* = linear regression coefficient.

† Multilevel binary logistic regression model was conducted.

‡ Multilevel linear regression model was conducted.

** *P* < .05.

### 3.5. The association in middle-aged and elder adults

An insignificant association between reading habit, depression category, and depression score was found in the middle-aged group aged from 41 to 65 years. However, among the elderly (aged >65 years), there was a significant negative association between reading habit, depression category, and depression score (see Table S1, Supplemental Digital Content, http://links.lww.com/MD/I244), which illustrated the association between reading habit and depression category, CES-D_20_ score with ORs/coefficient and 95% confidence intervals in different age group). Association between number of reading habit and depression did not show statistic significance both in middle-aged and elderly participants (see Table S2, Supplemental Digital Content, http://links.lww.com/MD/I245), which illustrated the association between number of reading habit and depression category, CES-D_20_ score with ORs/coefficient and 95% confidence intervals in different age group).

## 4. Discussion

In this study of a descriptive and nationally representative sample of middle-aged and the elderly, after adjusting confounders, a negative relationship between reading habit and depression was found among adults aged >40 in China, especially in the elderly, but there was no difference between the number of readings and depression. The prevalence of depression in the current study was 13.02%, which was lower than that found in another middle-aged and elderly population from the China Health and Retirement Longitudinal Study.^[22]^ The plausible explanation for this inconformity between the 2 prevalence may be that the measurement tools for depression in the 2 studies were dissimilar. In addition, an unequable association between reading behavior and depression in different age groups was observed. This finding indicated that the association was more pronounced among the elderly population than among middle-aged adults.

This study found that people with reading habits had lower rates of depression, which is consistent with the results of previous studies on the mental health of people.^[15,16]^ Reading behavior seems to improve the ability to solve mood problems. As a considerable alternative to deal with troublesome affairs in daily life, reading is effective in resolving conflicts, self-psychological adjustment, uplifting mood, and altering negative thoughts. Stress has been recognized to contribute to the development of depression.^[23]^ Research has shown that reading can effectively relieve pressure from work or family.^[24,25]^ A good bedtime reading habit can effectively improve the quality of sleep,^[26]^ thus reducing the risk of depression caused by poor sleep.^[27]^ Depression is often associated with deficiencies in social functioning.^[28]^ Lack of understanding of the minds of others may be an important reason for the defects of social function. The foundation of understanding others’ minds is the ability to understand and share their feelings, which is called empathy.^[29]^ Studies have shown that reading can effectively improve the ability of empathy.^[24,30]^ Therefore, reading may effectively prevent depression by improving social functioning. In addition, the health effects of reading persist long after we put down the book, with some studies suggesting that reductions in depressive symptoms in adults persist months or even years later.^[31]^ It can be seen that the effect of reading on people’s mental health is long-lasting and beneficial.

The results showed that compared with middle-aged people, the reading effect of the elderly is more obvious. Middle-aged people are committed to work and family affairs, which may reduce their reading time and association. The elderly might have more leisure time to spend on reading compared to the middle-aged population. Fuller found that reading prompted people to engage in more social activities, which promoted opportunities to communicate and share emotions with others.^[32]^ In addition, studies have shown that participation in social activities has a positive impact on the mental health of old people,^[33,34]^ hence causing a tight association. Another rational explanation might be that most elderly are retired and there is no work and life stress to counteract the effect of reading behavior.

Additionally, reading is known to be a long-term investment in oneself, which improves one’s outlook toward the world, life, and values.^[35]^ And our results showed that appropriate number of readings related with lower OR of repression and lower depression evaluate score, which supported reading might be beneficial for mental health directly. Notably, reading habits relate to educational achievement in Chinese traditional culture. Reading projects such as the China Central Television reader program aroused strong responses throughout the country and encouraged more people to read.

Although the current study examined the association between reading habit and depression using the Chinese national representative survey data, several limitations need to be addressed. First, the reading and depression data were obtained simultaneously in the 2016 survey waves, which limited the inference of causal relations. Second, there was no detailed information on the reading category that restricted the possible association of different types of reading on depression. Third, although a lot of potential influencing factors were considered in this study, some possibility factors were not included due to the secondary analysis of the open database that was subject to the purpose of the original study design. These factors included, cognitive impairment linked with depression.^[36]^ In future studies, perspective research designed with cognitive impairment as a key controlled variable needs to be considered. Despite these limitations, the present study provides valuable data, supporting the connection between reading habits and depression in mainland China. The national representative sample ensured the credibility of the results obtained.

In conclusion, the current study found that reading habit was negatively associated with the prevalence of depression and CES-D_20_ scores among elderly Chinese adults except in the middle-aged population. However, moderate reading over the course of a year was associated with a lower risk of depression and a lower CES-D_20_ score in middle-aged and elderly population. Further perspective studies need to be performed to verify the association as it may be a potential alternative for clinicians and public health.

## Acknowledgments

The authors sincerely thank the China Family Panel Studies (CFPS) for providing consent for data access, the Peking University for funding by means of the 985 Program, and the Institute of Social Science Survey of Peking University for assisting us in conducting this study.

## Author contributions

**Conceptualization:** Xiao Yang, Jiangping Li.

**Formal analysis:** Degong Pan, Zhiqin Hai.

**Methodology:** Xiao Yang, Shulan He.

**Supervision:** Jiangping Li.

**Validation:** Degong Pan, Zhiqin Hai, Xiao Yang, Shulan He, Xiaojun Li, Jiangping Li.

**Writing – original draft:** Degong Pan, Zhiqin Hai, Xiaojun Li.

**Writing – review & editing:** Degong Pan, Zhiqin Hai, Jiangping Li.

## Supplementary Material
