# Supplementary material for: Association between reading and depression in Chinese adults
Source: Medicine (Baltimore). 2022 Dec 23;101(51):e32486. doi: 10.1097/MD.0000000000032486 (PMC9794234; doi:10.1097/MD.0000000000032486)
Supplement: Supplementary file 1 [file medi-101-e32486-s001.pdf]

Table S1 Association between whether of reading habit and depression category, CES-D_{20} score with ORs/coefficient and 95% CIs in different age group

Model	Middle aged adults				Elder aged adults			
	Depression[1]		CES-D_{20} score[2]		Depression[1]		CES-D_{20} score[2]	
	OR	95%CI	β	95%CI	OR	95%CI	β	95%CI
Model0	0.580***	0.464, 0.723	-1.337***	-1.774, -0.900	0.328***	0.216, 0.498	-3.255***	-4.083, -2.427
Model1	0.869	0.678, 1.112	-0.093	-0.555,0.368	0.568**	0.362, 0.892	-1.447***	-2.328, -0.567
Model2	0.902	0.704, 1.154	0.083	-0.363,0.529	0.639*	0.401, 1.018	-1.232***	-2.087, -0.376
Model3	0.865	0.671, 1.115	-0.004	-0.422,0.414	0.554**	0.334, 0.917	-1.324***	-2.118, -0.531
Model4	0.875	0.672, 1.139	-0.018	-0.442,0.405	0.563**	0.327, 0.968	-1.298***	-2.105, -0.491

* P<0.1, ** P <0.05, *** P<0.01

OR=odds ratio, CI=confidence interval, β=linear regression coefficient;

Model 0 contains depression category; Model 1=Model0+demographic variables; Model 2=Model1+ socioeconomic level information; Model 3= Model2+ health related indexes; Model 4 is the full model that contains all confounders and predictors.

[1] Multilevel binary logistic regression model were conducted;

[2] Multilevel linear regression model were conducted.

Model1 to Model4, age was excluded.
