# Supplementary material for: Association between reading and depression in Chinese adults
Source: Medicine (Baltimore). 2022 Dec 23;101(51):e32486. doi: 10.1097/MD.0000000000032486 (PMC9794234; doi:10.1097/MD.0000000000032486)
Supplement: Supplementary file 2 [file medi-101-e32486-s002.pdf]

Table S2 Association between number of reading habit and depression category, CES-D_{20} score with ORs/coefficient and 95% CIs in different age group

Model	Middle aged adults				Elder aged adults			
	Depression[1]		CES-D_{20} score[2]		Depression[1]		CES-D_{20} score[2]	
	OR	95%CI	β	95%CI	OR	95%CI	β	95%CI
Model0	0.981	0.956, 1.007	-0.014	-0.033, 0.006	0.992	0.966, 1.018	-0.009	-0.038, -0.020
Model1	0.992	0.970 1.015	-0.004	-0.030, 0.021	0.995	0.971, 1.020	-0.002	-0.031, 0.028
Model2	0.997	0.974, 1.021	0.002	-0.022, 0.026	0.993	0.961, 1.027	0.000	-0.027, 0.027
Model3	0.997	0.976, 1.019	-0.004	-0.026, 0.019	0.986	0.956, 1.017	-0.006	-0.030, 0.018
Model4	1.001	0.983, 1.019	-0.003	-0.026, 0.019	0.989	0.962, 1.017	-0.004	-0.027, 0.020

* P<0.1, ** P <0.05, *** P<0.01

OR=odds ratio, CI=confidence interval, β=linear regression coefficient;

Model 0 contains depression category; Model 1=Model0+demographic variables; Model 2=Model1+ socioeconomic level information; Model 3= Model2+ health related indexes; Model 4 is the full model that contains all confounders and predictors.

[1] Multilevel binary logistic regression model were conducted;

[2] Multilevel linear regression model were conducted.

Model1 to Model4, age was excluded.
